# Supplementary material for: Influence of Magnetic Anisotropy on the Ground State of [CH3NH3]Fe(HCOO)3: Insights into the Improper Modulated Magnetic Structure
Source: Inorg Chem. 2025 Apr 9;64(15):7348–63. doi: 10.1021/acs.inorgchem.4c05404 (PMC12015817; doi:10.1021/acs.inorgchem.4c05404)
Supplement: Supplementary file 1 — ic4c05404_si_001.pdf [file ic4c05404_si_001.pdf]

Supporting Information

Influence of Magnetic Anisotropy on the Ground State of $[CH_3NH_3]Fe(HCOO)_3$: Insights into the Improper Modulated Magnetic Structure

Laura Cañadillas-Delgado,[a] Lidia Mazzuca,[a] Sanliang Ling,[b] Matthew J. Cliffe[c] and Oscar Fabelo[a]*

[a] Institut Laue-Langevin, 71 Avenue des Martyrs, CS 20156, 38042 Grenoble Cedex 9, France.

[b] Advanced Materials Research Group, Faculty of Engineering, University of Nottingham, University Park, Nottingham, NG7 2RD, UK.

[c] School of Chemistry, University Park, University of Nottingham, NG7 2RD, UK.

Corresponding author: lcd@ill.fr

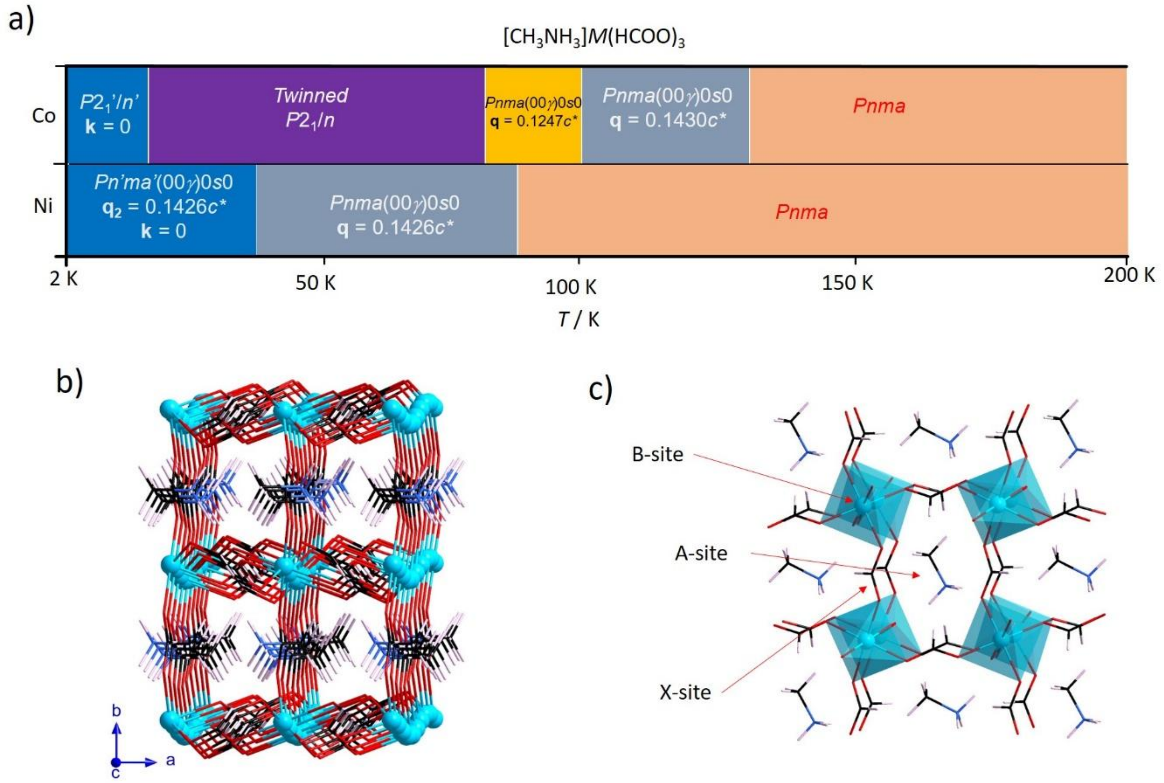

Figure S1. (**a**) Graphical representation of the different transition undergone by compounds **2** (Co) and **3** (Ni). (**b**) View of the modulated three-dimensional structure of compounds **2** (Co) and **3** (Ni), where it could be appreciated the displacement of the atoms mainly along the *b* axis. (**c**) View of the Perovskite-like framework. Carbon, oxygen, hydrogen, nitrogen and metal atoms are represented in black, red, pink, blue and light blue, respectively.

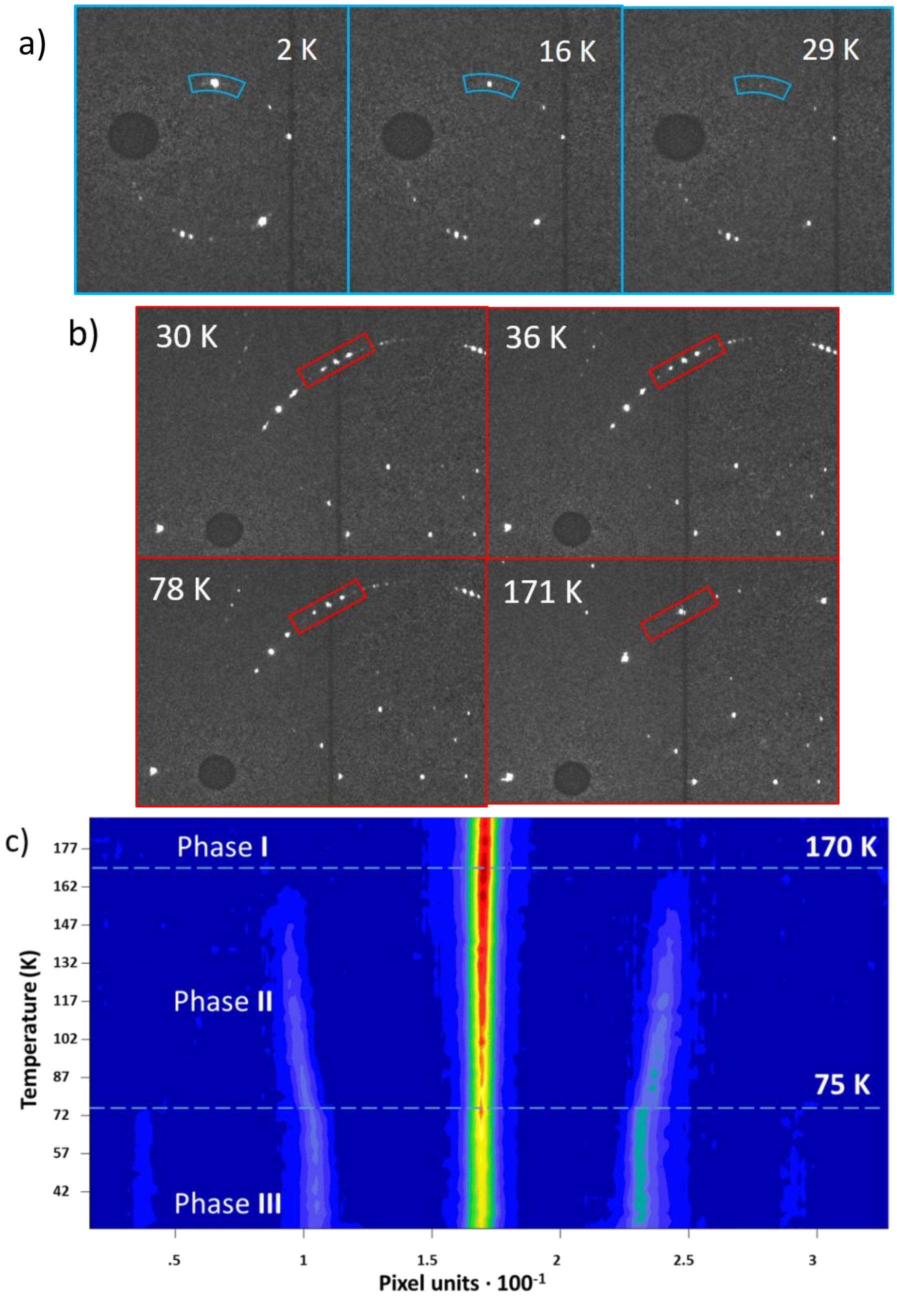

Figure S2. (**a-b**) Laue patterns of compound **1** (Fe) collected at different temperatures in the CYCLOPS diffractometer at ILL. Below 170 K new satellite reflections appear indicating the phase transition from phase **I** to phase **II**. Below 75 K, these satellite reflections move closer to the main reflection due to a second phase transition to phase **III**, which has a smaller **q** wave vector. Below 17 K new magnetic reflections appear revealing the onset of magnetic order. Note that during the warming process, at 30 K, the sample was reoriented to investigate a region of reciprocal space where satellite reflections are more visible. (**c**) Mesh plot of the temperature evolution that corresponds with the rectangle marked in red in the patterns between 30 K and 190 K.

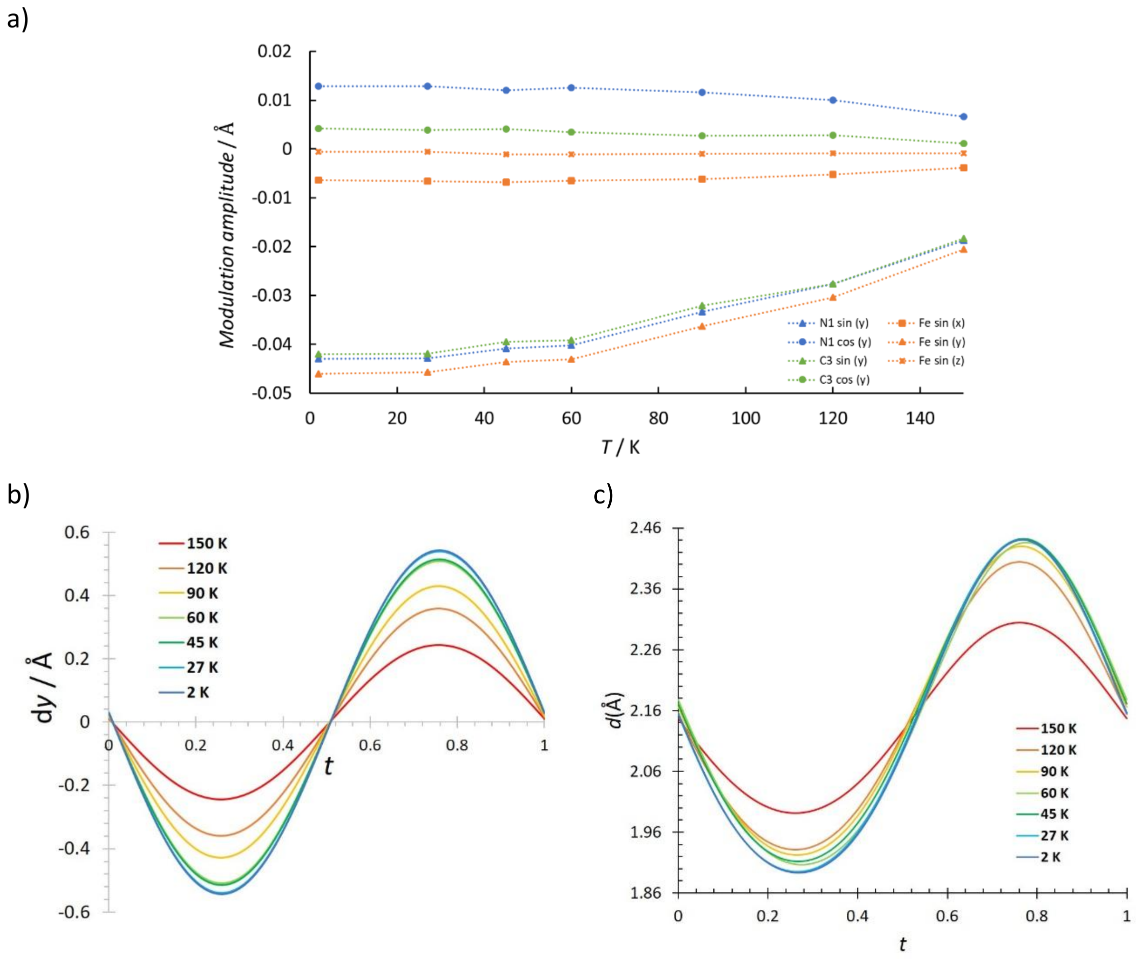

Figure S3. (**a**) The refined amplitude displacements in phases **II** and **III** for Fe(II) (orange), the C (green) and N (blue) atoms of the $(CH_3NH_3)^+$ counterion, representing the framework and the guest molecule, respectively. (**b**) Displacement along *b* axis of the iron atom and (**c**) H1N···O3 bond distances in the modulated phases of compound **1** (Fe) where it can be seen the effect of temperature.

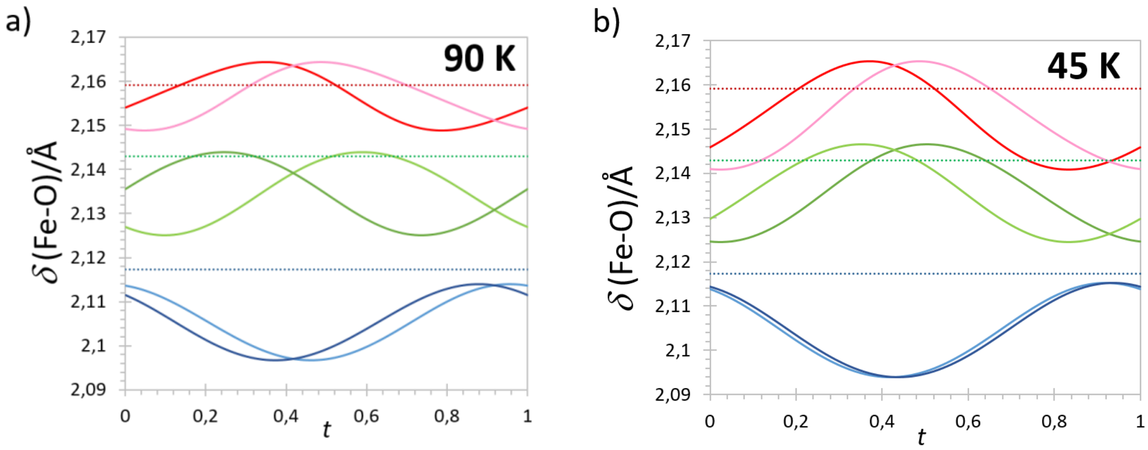

Figure S4. Modulation of the bond lengths between the iron and the oxygen atoms at 90 K (**a**) and 45 K (**b**) corresponding to phases **II** and **III,** respectively. The distances Fe1-O1, Fe1-O1a, Fe1-O2b, Fe1-O2c, Fe1-O3 and Fe1-O3a are represented in light blue, dark blue, red, pink, dark green and light green continuous lines, respectively. The distances Fe1-O1, Fe1-O2b and Fe1-O3 in the non-modulated phase **I** are represented in blue, red and green dotted lines, respectively. *Symmetry code*: *a* = -*x*, -*y*+1, -*z*+1; *b* = -*x*+1/2, -*y*+1, *z*-1/2; *c* = *x*-1/2, *y*, -*z*+3/2.

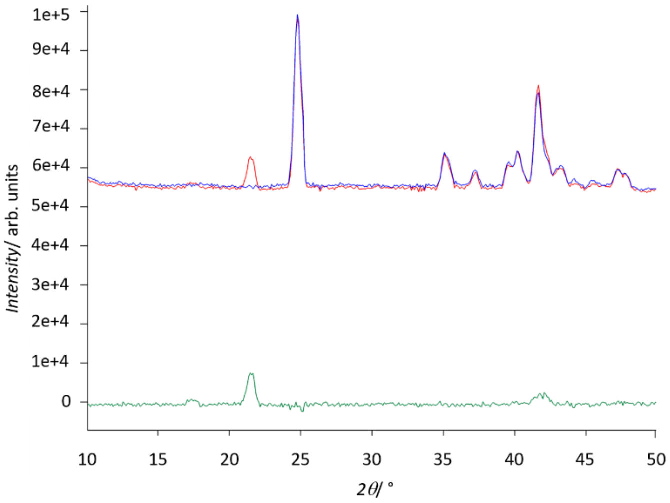

Figure S5. Neutron powder patterns of compound **1** (Fe) collected at 2 K (red) and 26 K (blue) using the high flux D1B diffractometer. The difference diffraction pattern has been represented as a solid green line.

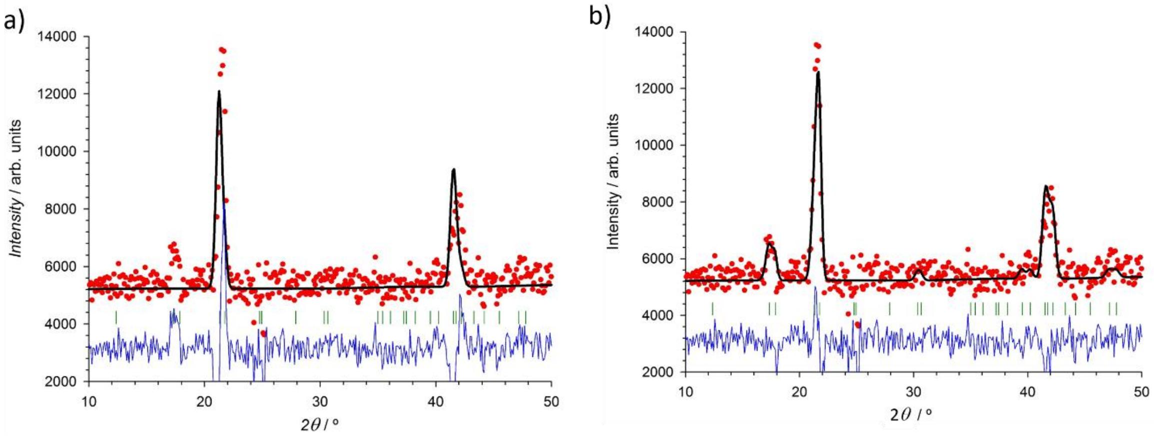

Figure S6. Fit of the difference pattern of compound **1** (Fe) in the *Pn'ma'* and in the *Pnma.*1 magnetic space groups, (a) and (b), respectively. Experimental data have been represented as red circles, calculated Rietveld patterns are shown as solid black lines, and difference between observed and calculated patterns have been plotted as solid blue lines. Vertical green marks represent the position of the Bragg reflections.

Table S1. List of the possible super space groups, obtained using ISODISTORT program,[1] compatible with the distortion modes obtained from considering the *Pnma* parent structure and **k** = (0, 0, 0) and $\mathbf{q_2}$ = 0.1425(2)c^* as modulation vectors for the magnetic and nuclear distortions, respectively.

Pnma.1(00*γ*)000, origin = (0,0,0,0)	*Pnma*.1(00*γ*)0*s*0, origin = (0,0,0,0)
Pmn2_1.1(00*γ*)000, origin = (1/4,3/4,0,0)	*Pmn*2_1.1(00*γ*)*s*0*s*, origin = (1/4,3/4,0,0)
Pn'm'a(00*γ*)000, origin = (0,0,0,0)	*Pn'm'a*(00*γ*)0*s*0, origin = (0,0,0,0)
Pm'n'2_1(00*γ*)000, origin = (1/4,3/4,0,0)	*Pm'n'*2_1(00*γ*)*s*0*s*, origin = (1/4,3/4,0,0)
Pnm'a'(00*γ*)000, origin = (0,0,0,0)	*Pnm'a'*(00*γ*)0*s*0, origin = (0,0,0,0)
Pm'n2_1'(00*γ*)000, origin = (1/4,3/4,0,0)	*Pm'n*2_1'(00*γ*)*s*0*s*, origin = (1/4,3/4,0,0)
Pn'ma'(00*γ*)000, origin = (0,0,0,0)	*Pn'ma'*(00*γ*)0*s*0, origin = (0,0,0,0)
Pmn'2_1'(00*γ*)000, origin = (1/4,3/4,0,0)	*Pmn'*2_1'(00*γ*)*s*0*s*, origin = (1/4,3/4,0,0)
Pnma.1(00*γ*)0*s*0, origin = (0,0,0,1/4)	*Pnma*.1(00*γ*)000, origin = (0,0,0,1/4)
Pmn2_1.1(00*γ*)*s*0*s*, origin = (1/4,3/4,0,0)	*Pmn*2_1.1(00*γ*)000, origin = (1/4,3/4,0,0)
Pn'm'a(00*γ*)0*s*0, origin = (0,0,0,1/4)	*Pn'm'a*(00*γ*)000, origin = (0,0,0,1/4)
Pm'n'2_1(00*γ*)*s*0*s*, origin = (1/4,3/4,0,0)	*Pm'n'*2_1(00*γ*)000, origin = (1/4,3/4,0,0)
Pnm'a'(00*γ*)0*s*0, origin = (0,0,0,1/4)	*Pnm'a'*(00*γ*)000, origin = (0,0,0,1/4)
Pm'n2_1'(00*γ*)*s*0*s*, origin = (1/4,3/4,0,0)	*Pm'n*2_1'(00*γ*)000, origin = (1/4,3/4,0,0)
Pn'ma'(00*γ*)0*s*0, origin = (0,0,0,1/4)	*Pn'ma'*(00*γ*)000, origin = (0,0,0,1/4)
Pmn'2_1'(00*γ*)*s*0*s*, origin = (1/4,3/4,0,0)	*Pmn'*2_1'(00*γ*)000, origin = (1/4,3/4,0,0)

(1) Campbell, B. J.; Stokes, H. T.; Tanner, D. E.; Hatch, D. M. *ISODISPLACE* : A Web-Based Tool for Exploring Structural Distortions. *J Appl Crystallogr* **2006**, *39* (4), 607–614. https://doi.org/10.1107/S0021889806014075.
